# Supplementary material for: The Beneficial Effect of Eco-Friendly Green Nanoparticles Using Garcinia mangostana Peel Extract against Pathogenicity of Listeria monocytogenes in Female BALB/c Mice
Source: Animals (Basel). 2020 Mar 29;10(4):573. doi: 10.3390/ani10040573 (PMC7222409; doi:10.3390/ani10040573)
Supplement: Supplementary file 1 [file animals-10-00573-s001.pdf]

Table S1. Content of total phenols, flavonoids, and antioxidant activity of flavonoids free-radical scavenging (DPPH and ABTS) and ferric reducing antioxidant power (FRAP) in methanol-extracted air- and freeze-dried mangosteen peel nanoparticles.

Name of sample	T. Phenols (mg gallic acid/g sample)	T. Flavonoids (mg catechin/g sample)	DPPH (%)	ABTS (g trolox/g sample)	FRAP (g trolox/g sample)
	M ± SD	M ± SD	M ± SD	M ± SD	M ± SD
Air-dried mangosteen peel	401.303 ± 14.498	1.057 ± 0.119	94.775 ± 0.122	13.075 ± 0.534	5.728 ± 0.152
Air-dried mangosteen peel nanoparticles	479.744 ± 2.564	4.711 ± 0.207	94.303 ± 0.074	16.069 ± 0.424	6.696 ± 0.085
Freeze-dried Mangosteen peel	815.311 ± 3.935	15.394 ± 1.496	94.643 ± 0.128	29.943 ± 0.058	12.982 ± 0.368
Freeze-dried mangosteen peel nanoparticles	1112.646 ± 1.842	14.154 ± 0.119	95.707 ± 0.070	42.753 ± 0.200	17.806 ± 0.056

Values are means of three replicates. Results were given as mean ± SD.

10^{10} CFU/ml

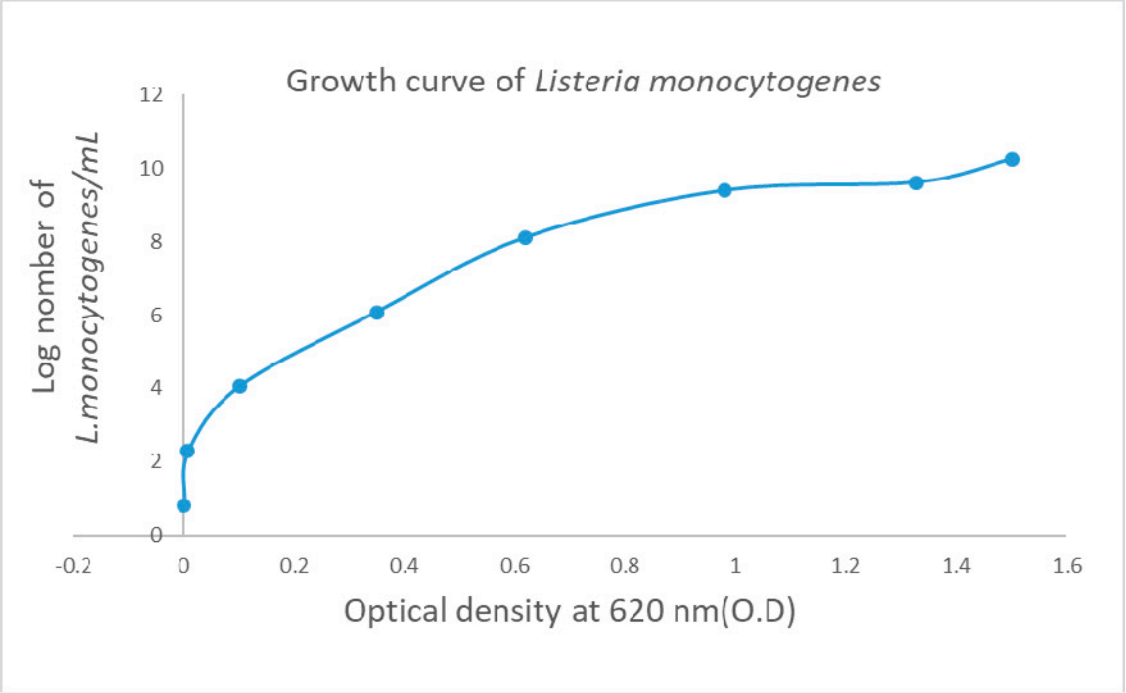

Figure S1. Total viable count of *Listeria monocytogenes* ATCC 19114 against Optical density at 620 nm.

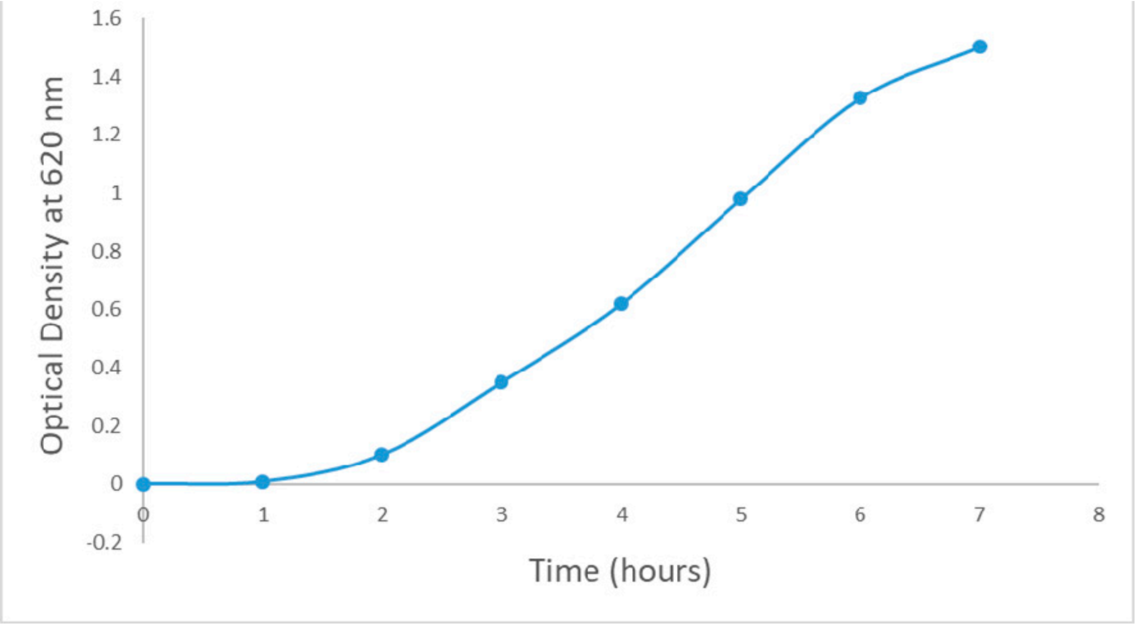

Figure S2: optical density of *Listeria monocytogenes* ATCC 19114 at 620 nm at different time per hours.
